# Effects of Exercise on Balance Function in People with Knee Osteoarthritis: A Systematic Review and Meta-Analysis of Randomized Controlled Trials

**DOI:** 10.3390/healthcare13111312

**Published:** 2025-06-01

**Authors:** Xingyue Wang, Zhuying Chen, Yin Liang, Hao Su, Tongling Wang, Yuanyuan Lv, Laikang Yu

**Affiliations:** 1 Beijing Key Laboratory of Sports Performance and Skill Assessment, Beijing Sport University, Beijing 100084, China; 15558541694@163.com; 2 China Basketball College, Beijing Sport University, Beijing 100084, China; 3 Department of Strength and Conditioning Assessment and Monitoring, Beijing Sport University, Beijing 100084, China; zhuying20232120126@126.com (Z.C.); 18076397318@163.com (Y.L.); 15662755237@163.com (H.S.); 4 Institute of Physical Education, Huzhou University, Huzhou 313000, China; 02942@zjhu.edu.cn; 5 China Institute of Sport and Health Science, Beijing Sport University, Beijing 100084, China

**Keywords:** exercise, knee osteoarthritis, balance function, multicomponent training

## Abstract

**Objectives**: This study sought to evaluate the impact of exercise on balance function in individuals with knee osteoarthritis (KOA) and determine the most effective exercise protocols for balance enhancement. **Methods**: A systematic literature search was performed across five major electronic databases (PubMed, Web of Science, EBSCO, Cochrane, Scopus) until 13 September 2024. Statistical synthesis was conducted using weighted mean differences (WMDs) with 95% confidence intervals under a random-effects model. **Results**: Analysis of 22 studies revealed significant improvements in balance function following exercise interventions. Outcomes measured by the Berg balance scale (BBS, WMD, 2.65, *p* < 0.00001) and timed up and go test (TUG, WMD, −0.59, *p* < 0.0001) demonstrated clinically relevant enhancements in KOA populations. Subgroup analyses revealed that multicomponent training (WMD, 6.25, *p* = 0.003), interventions lasting ≥ 8 weeks (WMD, 4.92, *p* = 0.002), sessions ≥ 60 min (WMD, 7.42, *p* = 0.002), frequency ≥ 3 times per week (WMD, 2.83, *p* = 0.0005), weekly time ≥ 180 min per week (WMD, 7.42, *p* = 0.002), and patients < 60 years (WMD, 6.71, *p* = 0.002) were associated with greater improvement in BBS. **Conclusions**: Exercise significantly improved balance function in KOA patients, with multicomponent training emerging as the most effective intervention. Based on the findings of this meta-analysis, clinicians should recommend that KOA patients engage in exercise at least three times per week, with each session lasting at least 60 min, to achieve a total weekly time of 180 min. These recommendations are particularly relevant for patients less than 60 years, who may experience greater benefits from exercise interventions.

## 1. Introduction

Knee osteoarthritis (KOA), a progressive joint disorder, exhibits strong age-dependent prevalence patterns. It is characterized by pathological changes such as cartilage degeneration, bone remodeling, osteophytic proliferation, and chronic synovitis. These degenerative cascades clinically present as persistent arthralgia, restricted range of motion, periarticular edema, and progressive functional impairment of the affected joint [1,2]. Epidemiological studies indicate a marked gender disparity, with female populations demonstrating significantly higher susceptibility compared to males [3,4]. The molecular mechanisms underlying osteoarthritis involve the progressive loss of articular chondrocytes due to cartilage damage, leading to reduced joint space, increased bone friction, and subsequent pain and restricted movement [5]. Compared to their healthy counterparts, individuals with KOA typically exhibit poorer physical function, including reduced lower limb muscle strength, impaired balance, and decreased mobility [6,7]. Balance deficits, in particular, have been identified as a significant contributor to mobility limitations and an elevated risk of falls in this population [8].

Exercise has been shown to improve lower limb muscle strength and both static and dynamic balance in older adults, including those at risk of falls [9]. Although cartilage damage in KOA is irreversible and cannot be fully reversed by medication, physiotherapy, or surgery [10], modifiable risk factors such as joint dysfunction and muscle weakness can be targeted through primary and secondary prevention strategies [11]. As a result, non-pharmacological interventions, particularly exercise, are considered central to the management of KOA, aiming to alleviate symptoms and enhance or maintain physical function [10,12,13].

Given the challenges posed by muscle weakness and pain in KOA patients, exercise interventions that address these issues are critical. Strength training, for instance, can mitigate muscle weakness by improving muscle mass and recruitment [14]. Clinical guidelines endorse exercise as a key component of non-pharmacological treatment due to its ease of implementation, low risk of adverse effects, and cost-effectiveness [3]. However, the effects of exercise on balance function in KOA patients remain inconsistent. For example, while a 6-week balance exercise program improved dynamic balance in KOA patients, no significant changes were observed in functional balance scores [15]. Similarly, Tai Chi has been shown to enhance balance function in the elderly with KOA patients, but the overall evidence remains mixed [16].

Falls are a major cause of unintentional injury and mortality among adults aged 65 and older, with balance impairment being a key predictor [17,18,19,20]. Standardized assessments such as the Berg balance scale (BBS) and timed up and go test (TUG) are widely used to evaluate balance function and identify functional limitations [21]. In KOA patients, the loss of balance, proprioception, and postural control can lead to reduced confidence in mobility and an increased risk of falls [22], ultimately compromising their ability to perform daily activities independently [23]. While some studies suggest that balance-based exercises can reduce fall risk in older adults [24], others indicate that balance training alone may not improve muscle strength [25]. Nevertheless, a randomized controlled trial (RCT) has demonstrated that physical activity training can mitigate the negative effects of KOA [26].

Despite the growing body of research, previous studies have often neglected to consider exercise modality, including intervention duration, frequency, session duration, and weekly time, leading to variability in outcomes. To address this gap, we conducted this study to evaluate the impact of exercise on balance function in KOA patients and to identify the optimal exercise modalities for improving balance function in this population.

## 2. Materials and Methods

This study was conducted in accordance with the Cochrane Handbook for Systematic Reviews of Interventions [27] and the Preferred Reporting Items for Systematic Evaluation and Meta-Analyses guidelines (PRISMA, 2020) [28]. The study protocol was registered with PROSPERO (CRD42024604651).

### 2.1. Search Strategy

A comprehensive search was performed across multiple electronic databases, including PubMed, Web of Science, EBSCO, Cochrane, and Scopus, up to 13 September 2024. The search strategy utilized a combination of keywords and Medical Subject Headings (MESH) terms such as “exercise”, “balance”, and “knee osteoarthritis” (Table S1). Additionally, the reference lists of identified systematic reviews and meta-analyses were manually screened to ensure no relevant studies were missed. Two independent authors conducted the search and screening process. Any discrepancies between the authors were resolved through discussion with a third author until consensus was reached.

### 2.2. Eligibility Criteria

The Population, Intervention, Comparison, Outcome (PICO) framework is used to define the inclusion: (a) Population: patients with KOA; (b) Intervention: randomized controlled trials (RCTs) with subjects randomly assigned to either the intervention or control group; (c) Comparison: studies that measured BBS or TUG at baseline and compared results post-intervention; (d) Outcome: the primary outcomes were balance function.

Exclusion criteria were (1) non-English publications; (2) reviews, conference articles, abstracts, or non-original research articles; (3) studies involving animal models; and (4) studies where the control group received any form of exercise intervention.

### 2.3. Data Extraction

Two authors independently extracted data from the included studies. The extracted information included (a) studies characteristics (first author’s name, publication year, sample size); (b) intervention details (intervention type, duration, frequency, session duration, weekly time); (c) participant characteristics (disease duration, age); and (d) pre- and post-intervention balance outcomes [mean and standard deviation (SD)]. In the event of disagreement, a third author was consulted to achieve consensus.

### 2.4. Methodological Quality Assessment

The methodological quality of the included studies was assessed using the Cochrane Risk of Bias 2 (RoB-2) tool. This tool evaluates domains such as selection bias, performance bias, detection bias, attrition bias, reporting bias, and other biases [29]. Two authors independently conducted the quality assessment, and any disagreements were resolved through discussion with the third author.

### 2.5. Statistical Analysis

Changes in mean and SD values for BBS and TUG were calculated for each study. When studies reported standard error (SE) or 95% confidence interval (CI), SD values were derived using established methods [27,30]. Data were pooled using the random-effects model to determine the weighted mean difference (WMD) and 95% CI. In case of high heterogeneity (I^2^ > 60%), meta-regression, subgroup analyses, and sensitivity analyses were conducted to explore potential sources of variability [31].

Subgroup analyses were performed based on intervention type (aerobic exercise, resistance exercise, multicomponent training), duration (<8 weeks, ≥8 weeks), frequency (<3 times per week, ≥3 times per week), session duration (<60 min per session, ≥60 min per session), weekly time (<180 min per week and ≥180 min), and participant age (<60 years, ≥60 years). Forest plots were generated using RevMan.5.4 software, while meta-regression, sensitivity analysis, and funnel plot were performed using Stata 17 software. Statistical significance was set at *p* < 0.05.

## 3. Results

### 3.1. Study Selection

**Figure 1:**
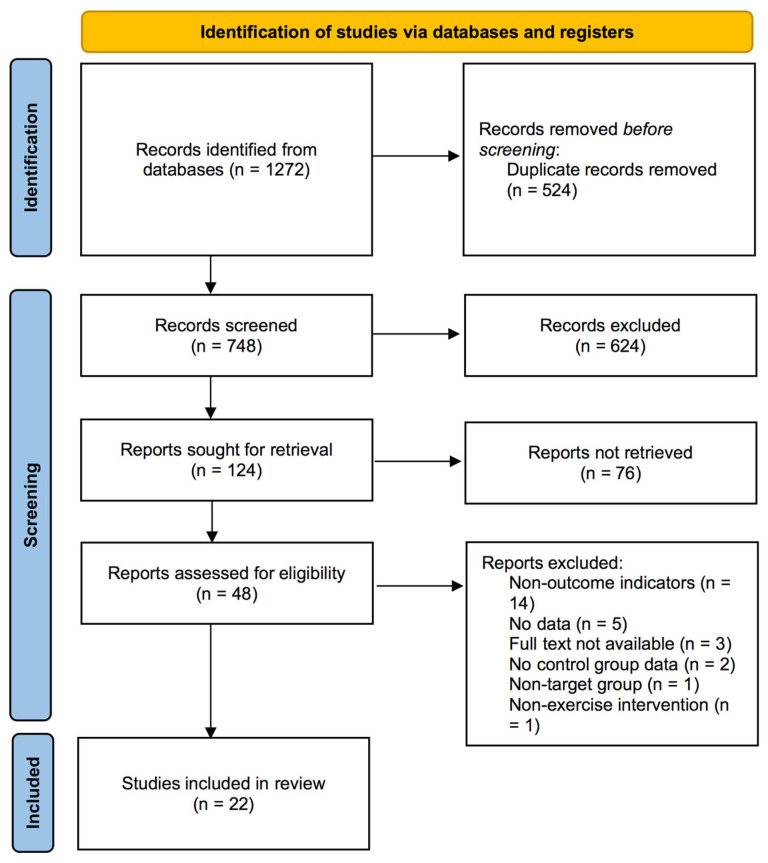
PRISMA flowchart of study selection.

The initial database search yielded 1272 records (Cochrane, *n* = 464; Scopus, *n* = 325; Web of Science, *n* = 313; Embase, *n* = 109; PubMed, *n* = 61). Following deduplication procedures, 748 records were retained for preliminary evaluation based on their titles and abstracts. Subsequent detailed assessment identified 48 studies that warranted comprehensive full-text examination. As shown in Table S1, after full-text assessment, 26 studies were excluded for the following reasons: (1) lack of relevant data (*n* = 5); (2) full text unavailable (*n* = 3); (3) absence of control group (*n* = 2); (4) non-target population (*n* = 1); (5) non-exercise interventions (*n* = 1); and (6) non-target outcome measures (*n* = 14). Finally, 22 studies [32,33,34,35,36,37,38,39,40,41,42,43,44,45,46,47,48,49,50,51,52,53] met the inclusion criteria. The complete workflow of this selection methodology is presented in Figure 1.

### 3.2. Characteristics of the Included Studies

The main characteristics of the included studies are summarized in Table S2. The experimental cohort comprised 31 intervention arms with 608 participants, contrasted by 22 control cohorts encompassing 526 participants. Therapeutic regimens across trials incorporated three principal modalities: aerobic exercise, resistance exercise, or multicomponent training. Implementation durations spanned from 14-day regimens to 24-week programs. Outcome measurement analysis revealed 10 studies employing BBS as the primary metric [32,33,34,35,36,37,38,39,40,41], complemented by 17 studies reporting TUG data [33,36,37,38,39,42,43,44,45,46,47,48,49,50,51,52,53]. Among the included studies, 7 focused exclusively on women [34,39,40,44,51,52,53], while 15 included both men and women [32,33,35,36,37,38,41,42,43,45,46,47,48,49,50]. Demographic characteristics indicated a mean participant age spectrum of 50.7–73.4 years, with all subjects having a radiologically confirmed KOA diagnosis. This cohort encompassed both pre- and post-total knee arthroplasty cases [36,45,46,47,49,52,53]. Of the 31 intervention groups, 1 involved aerobic exercise [50], 20 involved resistance exercise [32,33,34,35,36,38,42,46,47,48,51,52,53], and 9 involved multicomponent training [37,39,40,41,43,44,45,49]. Therapeutic administration frequency was maintained at 2–5 sessions weekly, with one trial omitting this parameter [45]. The mean session duration ranged from 15 to 90 min, with 5 studies not reporting session duration [33,35,44,46,47]. The total weekly time ranged from 75 to 270 min.

### 3.3. Risk of Bias

The methodological quality was evaluated through the RoB-2 tool, which stratifies bias risk into three tiers: low, high, and unclear. Analytical outcomes revealed a predominance of methodologically robust research, with seven studies demonstrating low bias risk, one exhibiting high risk, and the majority classified as having unclear risk levels (Figure S1).

### 3.4. Meta-Analysis Results

#### 3.4.1. Effects of Exercise on TUG in KOA Patients

**Figure 2:**
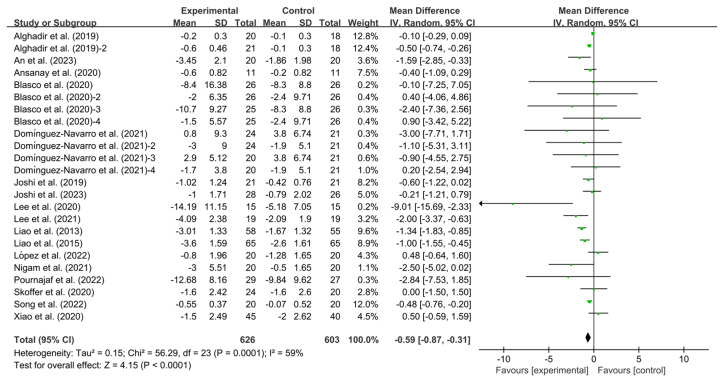
Meta-analysis results of the effect of exercise on TUG in KOA patients [33,36,37,38,39,42,43,44,45,46,47,48,49,50,51,52,53]. The pooled estimates were obtained from random effects analysis. Diamonds indicated the effect size of each study summarized as WMD. The size of the shaded squares was proportional to the percentage weight of each study. Horizontal lines represented the 95% CI and the vertical line represented the overall effect.

Twenty-four studies provided data on TUG. Exercise significantly improved TUG in KOA patients compared to controls (WMD, −0.59; 95% CI, −0.87 to −0.31, *p* < 0.0001, I^2^ = 59%, Figure 2).

#### 3.4.2. Effects of Exercise on BBS in KOA Patients

**Figure 3:**
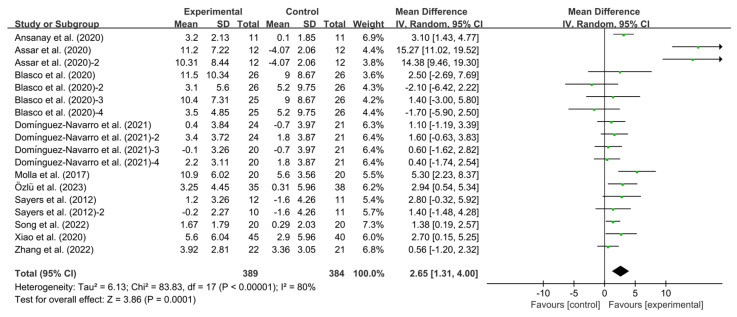
Meta-analysis results of the effect of exercise on BBS in KOA patients [32,33,34,35,36,37,38,39,40,41]. The pooled estimates were obtained from random effects analysis. Diamonds indicated the effect size of each study summarized as WMD. The size of the shaded squares was proportional to the percentage weight of each study. Horizontal lines represented the 95% CI and the vertical line represented the overall effect.

Exercise significantly improved BBS in KOA patients compared to controls (WMD, 2.65; 95% CI, 1.31 to 4.00, *p* < 0.00001, I^2^ = 80%, Figure 3).

Due to the high heterogeneity, subgroup analyses, meta-regression, and sensitivity analyses were conducted.

### 3.5. Subgroup Analysis

**Figure 4:**
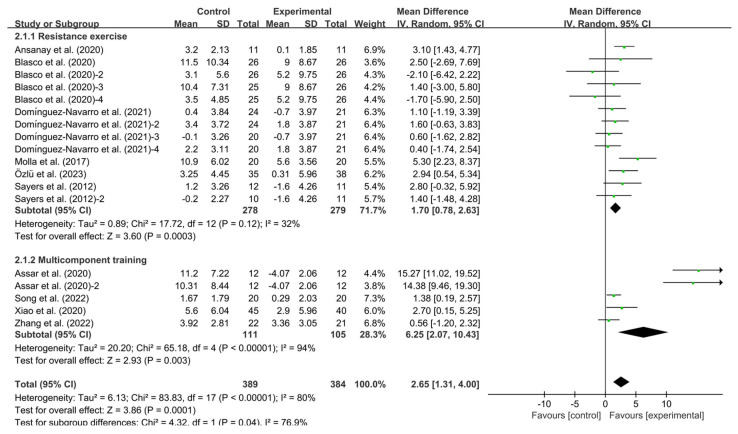
Meta-analysis results of the effect of types of intervention on BBS in KOA patients [32,33,34,35,36,38,39,40,41,42,43,44,45,46,47,48,49,51,52,53]. The pooled estimates were obtained from random effects analysis. Diamonds indicated the effect size of each study summarized as WMD. The size of the shaded squares was proportional to the percentage weight of each study. Horizontal lines represented the 95% CI and the vertical line represented the overall effect.

Resistance exercise (WMD, 1.70; 95% CI, 0.78 to 2.63, *p* = 0.003, I^2^ = 32%) and multicomponent training (WMD, 6.25; 95% CI, 2.07 to 10.43, *p* = 0.003, I^2^ = 94%, Figure 4) significantly improved BBS. Specifically, multicomponent training had a greater effect on improving BBS in KOA patients.

**Figure 5:**
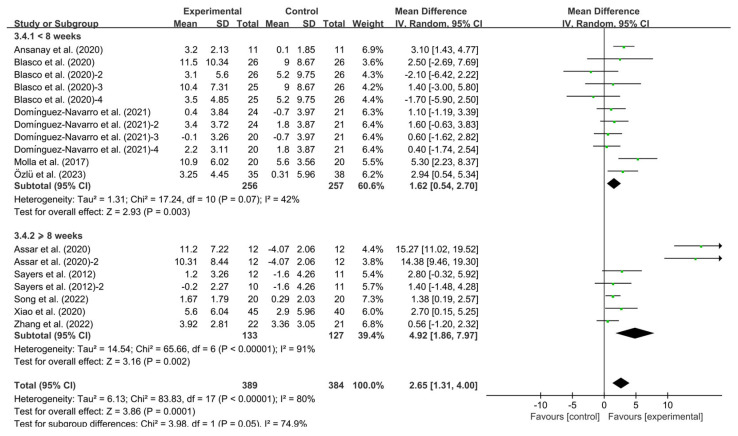
Meta-analysis results of the effect of intervention duration on BBS in KOA patients [32,33,34,35,36,37,38,39,40,41]. The pooled estimates were obtained from random effects analysis. Diamonds indicated the effect size of each study summarized as WMD. The size of the shaded squares was proportional to the percentage weight of each study. Horizontal lines represented the 95% CI and the vertical line represented the overall effect.

Additionally, analysis by intervention duration showed that interventions lasting <8 weeks (WMD, 1.62; 95% CI, 0.54 to 2.70, *p* = 0.003, I^2^ = 42%,) and ≥8 weeks (WMD, 4.92; 95% CI, 1.86 to 7.97, *p* = 0.002, I^2^ = 91%, Figure 5) significantly improved BBS. Specifically, interventions lasting ≥8 weeks had a greater effect on improving BBS in KOA patients.

**Figure 6:**
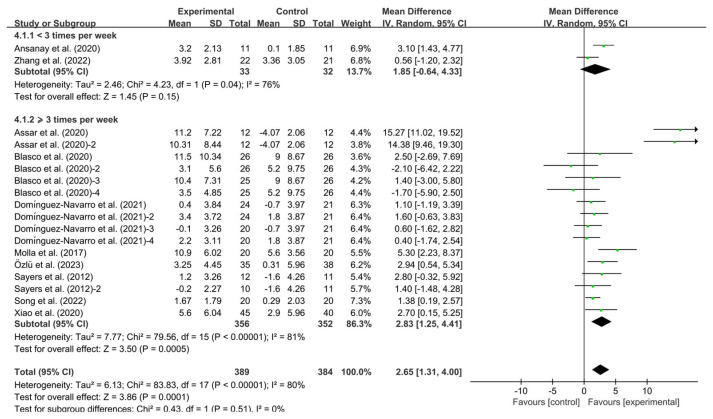
Meta-analysis results of the effect of frequency of intervention on BBS in KOA patients [32,33,34,35,36,37,38,39,40,41]. The pooled estimates were obtained from random effects analysis. Diamonds indicated the effect size of each study summarized as WMD. The size of the shaded squares was proportional to the percentage weight of each study. Horizontal lines represented the 95% CI and the vertical line represented the overall effect.

Additionally, interventions performed ≥3 times per week demonstrated significant enhancements in BBS (WMD, 2.83; 95% CI, 1.25 to 4.41, *p* = 0.0005, I^2^ = 81%, Figure 6). Conversely, interventions performed <3 times per week failed to achieve significant BBS improvements in KOA patients (WMD, 1.85; 95% CI, −0.64 to 4.33, *p* = 0.15, I^2^ = 76%, Figure 6).

**Figure 7:**
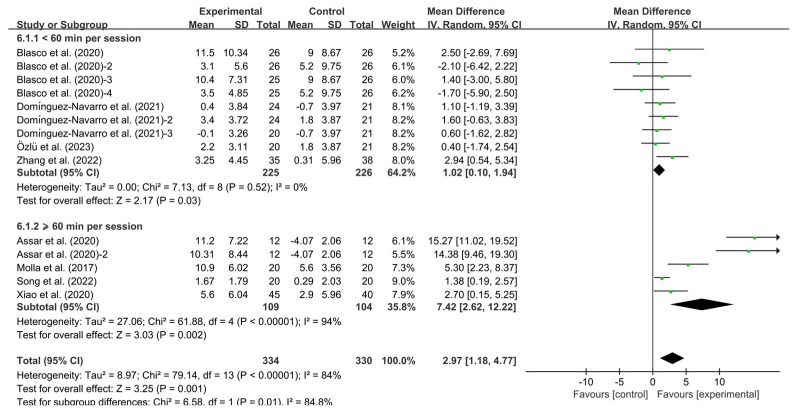
Meta-analysis results of the effect of duration of intervention per session on BBS in KOA patients [32,34,36,37,38,39,40,41]. The pooled estimates were obtained from random effects analysis. Diamonds indicated the effect size of each study summarized as WMD. The size of the shaded squares was proportional to the percentage weight of each study. Horizontal lines represented the 95% CI and the vertical line represented the overall effect.

Furthermore, both shorter (<60 min per session; WMD, 1.02; 95% CI, 0.10 to 1.94, *p* = 0.03, I^2^ = 0%) and extended (≥60 min per session; WMD, 7.42; 95% CI, 2.62 to 12.22, *p* = 0.002, I^2^ = 94%, Figure 7) significantly improved BBS. Specifically, prolonged session durations exhibited substantially greater balance enhancement effects compared to shorter protocols.

**Figure 8:**
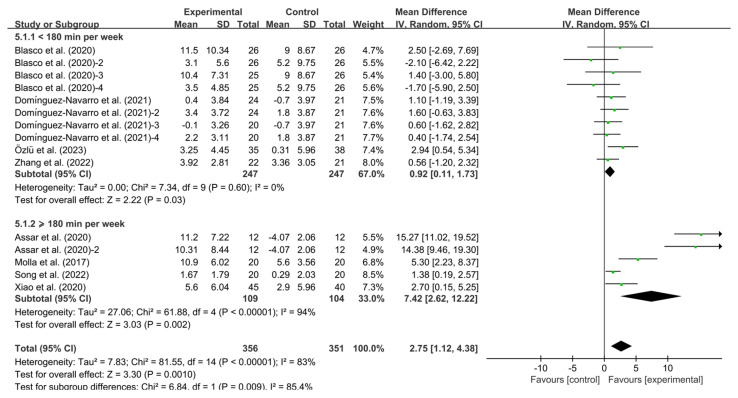
Meta-analysis results of the effect of duration of intervention per week on BBS in KOA patients [32,34,36,37,38,39,40,41]. The pooled estimates were obtained from random effects analysis. Diamonds indicated the effect size of each study summarized as WMD. The size of the shaded squares was proportional to the percentage weight of each study. Horizontal lines represented the 95% CI and the vertical line represented the overall effect.

Moreover, both sub-threshold (<180 min per week; WMD, 0.92; 95% CI, 0.11 to 1.73, *p* = 0.03, I^2^ = 0%) and supra-threshold (≥180 min; WMD, 7.42; 95% CI, 2.62 to 12.22, *p* = 0.002, I^2^ = 94%, Figure 8) regimens demonstrating significant BBS improvements. Notably, protocols exceeding the 180 min weekly threshold generated greater effects compared to suboptimal dosing regimens.

**Figure 9:**
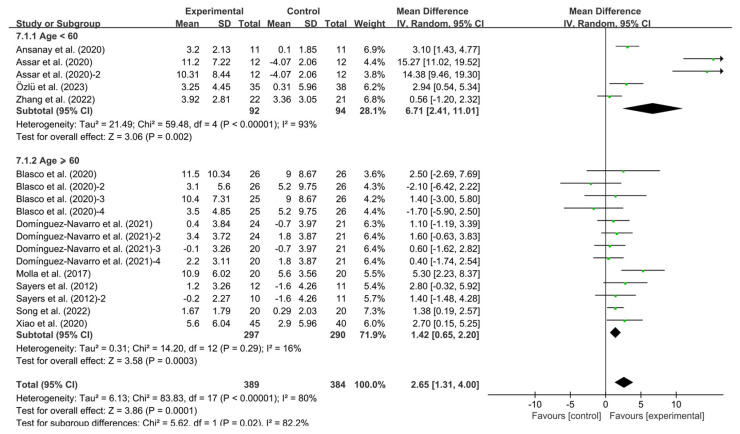
Meta-analysis results of the effect of different age groups on BBS in KOA patients [32,33,34,35,36,37,38,39,40,41]. The pooled estimates were obtained from random effects analysis. Diamonds indicated the effect size of each study summarized as WMD. The size of the shaded squares was proportional to the percentage weight of each study. Horizontal lines represented the 95% CI and the vertical line represented the overall effect.

Finally, exercise significantly improved BBS in KOA patients aged <60 years (WMD, 6.71, 95% CI, 2.4 to11.01, *p* = 0.002, I^2^ = 93%) and ≥60 years (WMD, 1.42, 95% CI, 0.65 to 2.20, *p* = 0.0003, I^2^ = 16%, Figure 9). Specifically, exercise had a greater effect on improving BBS in KOA patients aged <60 years.

### 3.6. Meta-Regression

As shown in Table S2, no significant correlations were found between intervention duration (*p* = 0.631) or frequency (*p* = 0.852) and BBS. However, significant associations were observed for session duration (*p* = 0.016), weekly time (*p* = 0.022), and participant age (*p* = 0.049).

### 3.7. Publication Bias

Funnel plot morphometric evaluation demonstrated skewed distributions in both balance outcomes (Figures S2 and S3). Egger’s test confirmed the absence of small-study effects, with intercept deviations remaining within null hypothesis thresholds (BBS, *p* = 0.134; TUG, *p* = 0.084; Table S3).

### 3.8. Sensitivity Analysis

Sensitivity analyses demonstrated that the overall effect of exercise on BBS (Figure S4) and TUG (Figure S5) in KOA patients remained consistent in direction and significant when any single study was excluded.

## 4. Discussion

### 4.1. Main Findings

The systematic evaluation of 22 RCTs demonstrated that exercise produced significant enhancements in balance function, evidenced by improvements in BBS and TUG. Subgroup analyses revealed that multicomponent training, interventions lasting ≥8 weeks, sessions performed ≥3 times per week, session durations ≥60 min, and weekly time ≥180 min were particularly effective in improving BBS. Additionally, patients aged <60 years showed greater improvements in balance function.

### 4.2. Effects of Exercise on Balance Function in KOA Patients

The findings of this study align with existing evidence supporting exercise as a core component of non-pharmacological management for KOA. Exercise interventions resulted in an overall improvement of 2.65 (WMD) in BBS and a reduction of 0.59 (WMD) in TUG, highlighting their efficacy in enhancing postural stability and balance control. These results are consistent with recommendations from the European Alliance of Associations for Rheumatology (EULAR) [54] and the Hong Kong League Against Osteoarthritis (LLOA) guidelines [55], which advocate for exercise as a first-line treatment for KOA.

The results of a previous study indicated that massage, as one of the non-pharmacological treatments, can improve pain indices and physical function in KOA patients; however, its long-term effects and sustained benefits remain unknown [56]. Although there is insufficient evidence to recommend one form of exercise over another [2], it is reasonable to advocate for exercise as a core treatment for KOA patients. Additionally, when balance function declines, it is accompanied by a decrease in physical function and quality of life, as well as a reduction in the patient’s confidence in engaging in activities or exercise. Gunn et al. [57] found that kinesiophobia is common in symptomatic KOA patients, which may hinder physical activity as patients avoid participating in general exercise that they perceive as “harmful” to their joints. Furthermore, Alghadir et al. [50] demonstrated that retrograde ambulation training significantly enhances quadriceps neuromuscular performance while concurrently reducing pain severity and functional limitations. When compared with healthy individuals, KOA patients have decreased muscle function and functional capacity, and decreased quadriceps strength is a risk factor for KOA patients [7,58]. Previous studies have shown that an increased risk of KOA is associated with weakness of the knee extensor muscles [14,59]. Moreover, quadriceps exercises aim to augment knee joint stability through selective muscle recruitment patterns [60], thereby improving balance performance and reducing fear or avoidance of exercise in KOA patients. It has been shown that exercise can increase the strength of the lower limbs in KOA patients, which reduces internal knee forces, decreases pain, and improves balance function [14].

### 4.3. Effects of Different Exercise Modalities on Balance Function in KOA Patients

The subgroup analysis provided valuable insights into the optimal exercise modalities for improving balance in KOA patients. According to the intervention type, the majority of interventions for KOA patients aim to enhance postural stability and balance during exercise. Contemporary therapeutic approaches predominantly incorporate three core modalities: aerobic exercise, resistance exercise, and multicomponent training. Previous studies confirm consistent associations between these exercise paradigms and postural stability enhancement, irrespective of intervention specificity [6,61]. Both resistance exercise and multicomponent training have been shown to improve balance function in KOA patients. Our findings indicated that multicomponent training is more beneficial for KOA patients compared to resistance exercise. The benefits of aerobic exercise primarily stem from an increase in oxidative capacity, and when combined with resistance exercise, it significantly enhances balance. A previous meta-analysis suggested that an optimal exercise program for KOA patients should have a singular goal and focus on specific physiological adaptations—whether aerobic capacity, quadriceps torque generation, or neuromuscular coordination [62]. This aligns with our recommendation for multicomponent training.

Previous studies have demonstrated that resistance exercise improves functional capacity and reduces pain in KOA patients [63]. However, Ansanay et al. [33] found that the addition of dexterity and perturbation training to resistance exercises was more effective in improving balance function in KOA patients than resistance exercises alone. Meanwhile, Sadeghi et al. [64] observed that mixed exercise, virtual reality-based game exercise, and balance training significantly improved leg strength and balance in older men, with multicomponent training exhibiting a clinically meaningful superior improvement over the other intervention groups. This is consistent with our results, which indicated that multicomponent training resulted in more significant improvements than resistance exercise. Numerous recommendations, including those of EULAR [54], affirm that combination therapy significantly improves balance function more than monotherapy, and one of the core recommendations is that individualized, multicomponent training management programs should be provided to KOA patients.

Intervention duration played a critical role in determining outcomes. Our results showed that ≥8 weeks of exercise was more effective than <8 weeks of exercise in improving balance function in KOA patients, which aligns with previous studies. Husby et al. [65] showed that 8 weeks of resistance exercise after total knee replacement surgery could maintain the sustained gains from the intervention at the 12-month post-intervention follow-up, as could an additional 8 weeks of strength training. In addition, the results of an RCT comparing the efficacy of Tai Chi and other physical therapies revealed that the benefits of Tai Chi were maintained for up to 52 weeks after a 12-week intervention [66]. Furthermore, Chen et al. [6] documented significant functional gains following three-month exercise regimens in geriatric KOA cohorts. However, Chaipinyo et al. [67] showed that a 4-week home resistance exercise and balance training had no significant improvement in pain and flexibility in KOA patients, which raises the question of whether the lack of improvement is associated with the insufficient intervention duration. Therefore, for KOA patients aiming to better alleviate pain and improve balance, an intervention duration of ≥8 weeks appears to offer more benefits and maintain good functional performance for a relatively long period after the intervention.

Similarly, our results demonstrated that exercising at least three times per week is more effective for improving balance function in KOA patients, which aligns with a previous study, showing that, for optimal results, exercise programs should be supervised and conducted three times a week [62]. According to the American College of Sports Medicine (ACSM) guideline recommendations, resistance exercise should be performed three times a week in elderly patients [68]. Based on our findings, resistance exercise combined with other exercise types is the most suitable exercise for KOA patients to improve balance function, especially when focusing on strengthening the lower limbs, which is most effective at a frequency of three times per week or more. Additionally, to enhance the functional abilities needed for daily life, it is crucial to prioritize balance, strength, and proprioception, necessitating a variety of rehabilitation exercises to restore functional performance [69]. Moreover, Lee et al. [53] showed that progressive dynamic balance training performed five times per week significantly improves balance and quality of life in elderly women who underwent a total knee arthroplasty.

Considering that the subjects were recent surgical patients, simply increasing the duration of a single intervention may not yield better results, as KOA patients or the postoperative population often experience low muscle strength, and over-exercise can lead to fatigue, negatively impacting the effectiveness of the intervention and fostering negative attitudes towards exercise. The results of a trial involving combined balance exercises performed three times per week showed beneficial effects on both static and dynamic balance in individuals following total knee replacement [61].

We cannot solely attribute this improved outcome to three interventions a week. Our results suggested that session duration ≥60 min led to better outcomes. Both healthy older adults and special populations seem to experience greater gains with a session duration of 60 min or more. A previous study demonstrated that multicomponent training significantly improved balance function in healthy older adults who performed approximately 60 min of exercise per session [70]. In addition, a Tai Chi exercise program with 60 min sessions showed improvement in balance function in KOA patients [71]. Furthermore, Mihalko et al. [72] found that a multicomponent training program of 60 min, three times a week, improved balance in adults with overweight KOA. However, longer interventions, such as those exceeding 90 min, should be carefully selected and verified by further experimental studies to determine their potential benefits for KOA patients.

Weekly time also influenced outcomes, with interventions totaling ≥180 min per week showing significant improvements in balance function in KOA patients, aligning with previous studies. The results of an aquatic exercise training study revealed that a weekly 180 min intervention significantly improved lower limb muscle weakness in women with KOA [73]. Since the balance function is closely associated with quadriceps strength, a longer intervention period or cycle can lead to greater muscular gains, enhancing patients’ confidence through improved muscle strength and size. Additionally, Kim et al. [74] showed that a weekly 180 min intervention involving stabilizing and balancing exercises was effective in stimulating the deep stabilizing muscles and proprioception, ultimately improving balance function. This improvement harmonized the muscles around the knee joint, reducing joint load and restoring muscle function. Combined with the above findings, it is evident that, for KOA patients, achieving more than 180 min of exercise per week necessitates both an increase in the frequency of exercise and a reasonable extension of the session duration.

Age was another important factor; patients <60 years showed greater improvements in balance function compared to older adults. The older population with KOA already exhibits weakened lower limb strength, necessitating gradual adaptation to exercise, as rushing may lead to ineffective or detrimental outcomes. A previous review showed that individuals over 40 years of age had a 2% higher risk of developing KOA compared to those under 40 [75]. KOA progressively worsens with age [76], and early intervention, whether during disease progression or post-surgery, can effectively slow the progression of symptoms [75]. A previous study showed that age was significantly negatively correlated with quadriceps thickness but positively correlated with balance function; thus, advancing age leads to increased muscle atrophy, which subsequently impairs balance function in KOA patients [76]. Additionally, a meta-analysis found that, while physical activity positively impacts healthy aging in middle-aged and older adults, this effect diminishes over time [77]. As age increases, KOA patients exhibit inadequate muscle function and peripheral nerve responses, heightened joint instability and ligament laxity, as well as a slower anabolic response to growth factors [78], potentially reducing their sensitivity to exercise at the same intensity.

Considering the substantial evidence that highlights the benefits of exercise in reducing pain and enhancing balance function, KOA patients should be encouraged to incorporate exercise as a key component of their treatment plan. Personal preference, accessibility, and affordability may influence the most suitable approach for individual patients [2]. Therefore, based on the advice of their physician or rehabilitation practitioner, KOA patients should select multicomponent training that suits them, along with the appropriate intervention duration, tailored to their specific circumstances.

### 4.4. Strengths and Limitations

Three studies [16,79,80] investigated the effects of exercise on balance function (BBS and/or TUG) in KOA patients. However, these studies share a critical limitation: they do not differentiate between exercise modalities. Specifically, aerobic exercise, resistance exercise, and multicomponent training were analyzed collectively, which obscures the specific benefits of each intervention type. While existing evidence supports the general association between exercise and improved balance function in KOA patients, these studies fail to provide actionable guidance for clinical practice. For instance, they do not clarify whether aerobic exercise, resistance exercise, or multicomponent training is more effective. Furthermore, key parameters such as optimal session duration, weekly frequency, and total weekly exercise volume remain unaddressed. These gaps underscore the necessity of our study, which aims to deliver detailed, evidence-based exercise prescriptions tailored to balance improvement in KOA patients.

This study advances the existing literature by addressing three key strengths. First, we provide a comprehensive evaluation of how distinct exercise protocols influence balance function in KOA patients, offering specific recommendations for optimizing exercise prescriptions. Additionally, the inclusion of subgroup analyses allows us to identify specific exercise parameters, such as intervention duration, session duration, frequency, and weekly time, that are most effective in improving balance function. This provides valuable insights for clinical practice. Finally, our results demonstrate that multicomponent training confers the greatest balance benefits, providing clear clinical guidance for exercise prescription in this population.

This study has several limitations. First, the included studies did not differentiate patients who had undergone total knee arthroplasty and those who had not, which may have influenced the results. Second, some included studies lacked detailed information on exercise intensity, limiting the ability to assess their impact on outcomes. Third, the heterogeneity in exercise modalities and intervention details across studies may have contributed to variability in the results. Moreover, we recognize the importance of the minimal clinically important difference (MCID) in interpreting the clinical relevance of our results. However, it is crucial to note that there is currently no gold standard for MCID values specific to balance function (BBS and TUG) in KOA patients. Although previous studies reported the MCID values for KOA patients [81,82], these values do not directly apply to our research due to differences in participant characteristics, such as surgical status and disease grading. These differences can significantly impact the determination of MCID values. Finally, the subjective nature of quality assessment and the significant heterogeneity observed in the meta-analysis necessitate cautious interpretation of the findings.

## 5. Conclusions

Exercise significantly improved balance function in KOA patients, with multicomponent training emerging as the most effective intervention. The synthesis of evidence from this meta-analysis supports the implementation of structured exercise protocols for managing KOA, advocating regimens that include at least three weekly sessions of at least 60 min each. This prescription framework aims to achieve a cumulative weekly exercise volume of 180 min to optimize neuromuscular adaptation and biomechanical stability in affected populations. These recommendations are particularly relevant for patients less than 60 years, who may experience greater benefits from exercise interventions. Future research should focus on standardizing exercise protocols and exploring the long-term effects of different exercise modalities on balance and functional outcomes in KOA patients.

## Data Availability

All data generated or analyzed during this study are included in the article/Supplementary Material.

## Supplementary Materials

The following supporting information can be downloaded at: https://www.mdpi.com/article/10.3390/healthcare13111312/s1, Figure S1: Results of Cochrane risk of bias tool; Figure S2: Funnel plot of BBS; Figure S3: Funnel plot of TUG; Figure S4: Sensitivity analysis of BBS; Figure S5: Sensitivity analysis of TUG; Table S1. Search strategies; Table S2. Excluded studies list; Table S3: Characteristics of studies included in this meta-analysis; Table S4: Results of meta-regression; Table S5: Results of Egger’s test.

## Abbreviations

The following abbreviations are used in this manuscript:

KOA	Knee osteoarthritis
BBS	Berg balance scale
TUG	Timed up and go test
RCT	Randomized controlled trial
PRISMA	Preferred Reporting Items for Systematic Evaluation and Meta-Analyses
MESH	Medical Subject Headings
SD	Standard deviation
SE	Standard error
CI	Confidence interval
WMD	Weighted mean difference
EULAR	European Alliance of Associations for Rheumatology
LLOA	Hong Kong League Against Osteoarthritis
ACSM	American College of Sports Medicine
