# Supplementary material for: Effects of Exercise on Balance Function in People with Knee Osteoarthritis: A Systematic Review and Meta-Analysis of Randomized Controlled Trials
Source: Healthcare (Basel). 2025 Jun 1;13(11):1312. doi: 10.3390/healthcare13111312 (PMC12155419; doi:10.3390/healthcare13111312)
Supplement: Supplementary file 1 [file healthcare-13-01312-s001.zip › healthcare-3582513-supplementary.pdf]

Supplemental material

Effects of exercise on balance function in people with knee osteoarthritis: a systematic review and meta-analysis of randomized controlled trials

Figure S1. Results of Cochrane risk of bias tool. 2

Figure S2. Funnel plot of BBS 3

Figure S3. Funnel plot of TUG 4

Figure S4. Sensitivity analysis of BBS 5

Figure S5. Sensitivity analysis of TUG 6

Table S1. Search strategies 7

Table S2. Excluded studies list 8

Table S3. Characteristics of studies included in this meta-analysis 10

Table S4. Results of meta-regression 16

Table S5. Results of Egger's test 17

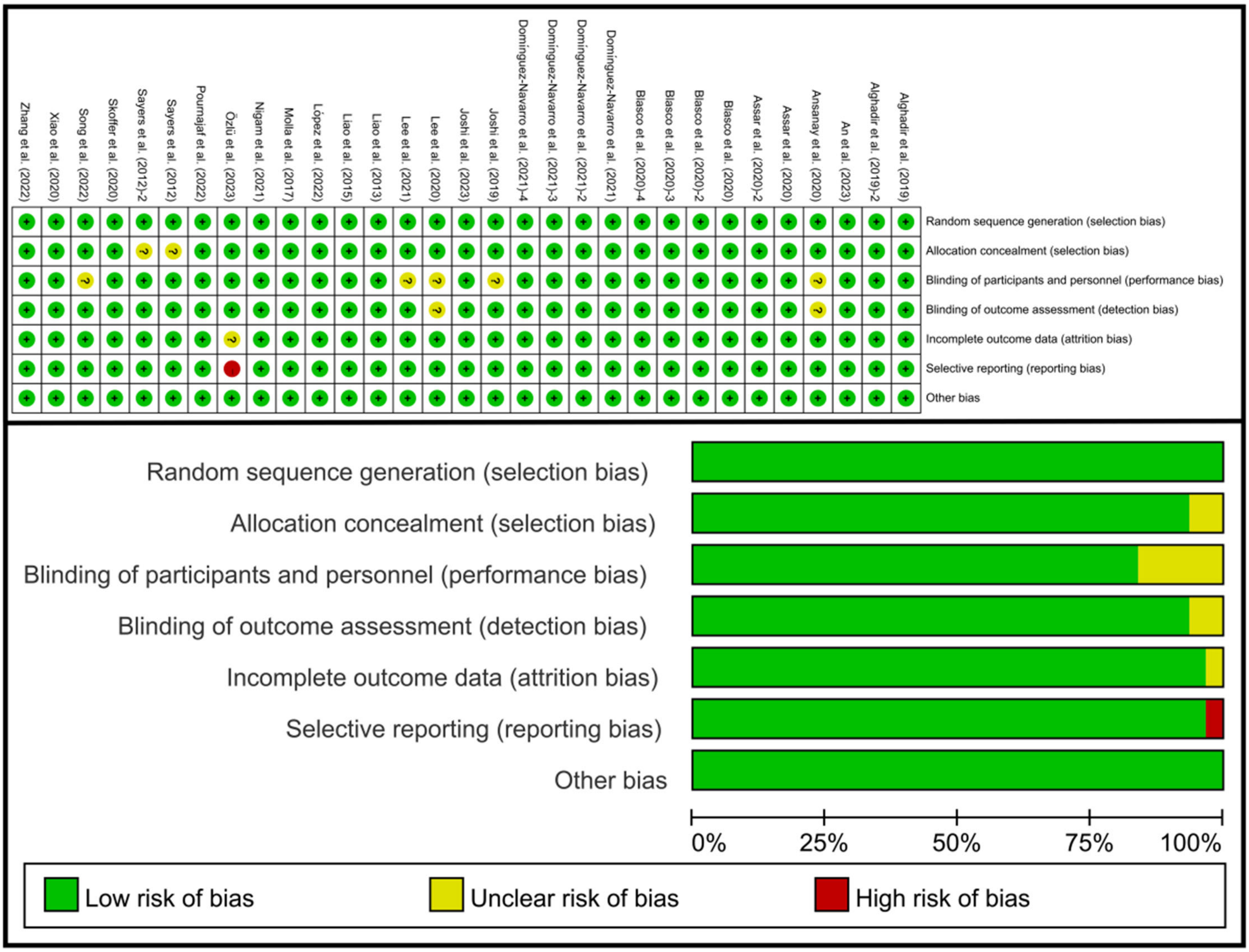

Figure S1. Results of Cochrane risk of bias tool.

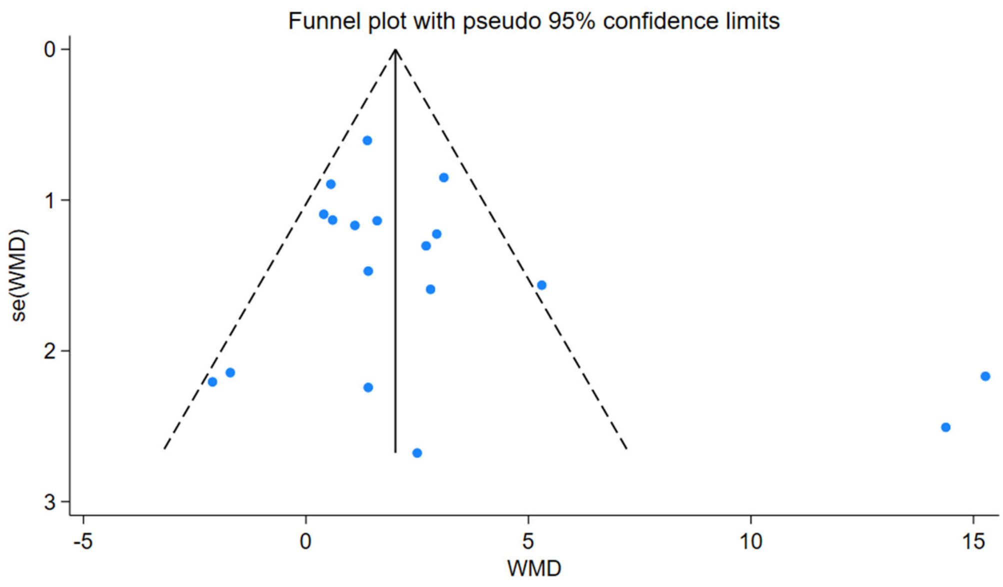

Figure S2. Funnel plot of BBS.

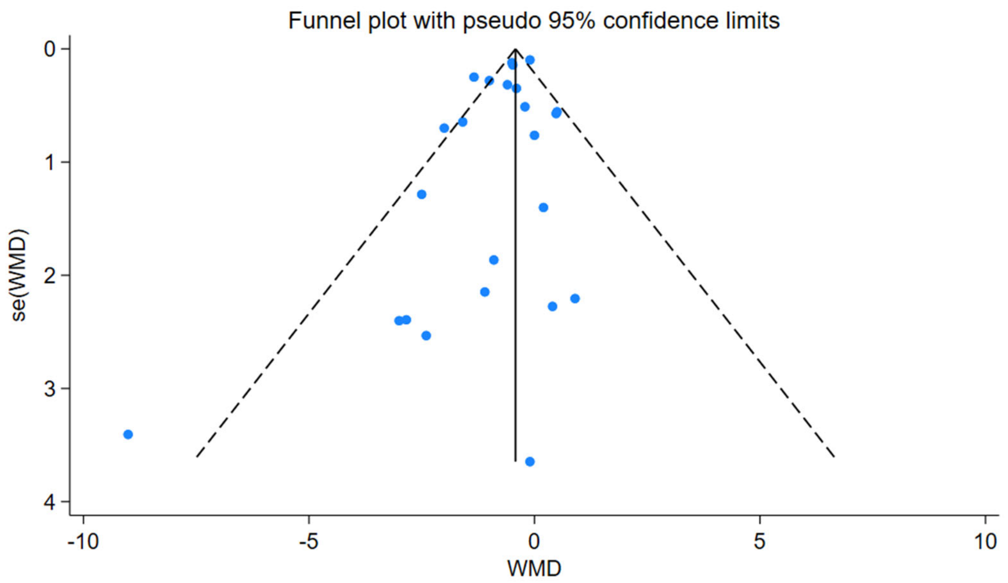

Figure S3. Funnel plot of TUG.

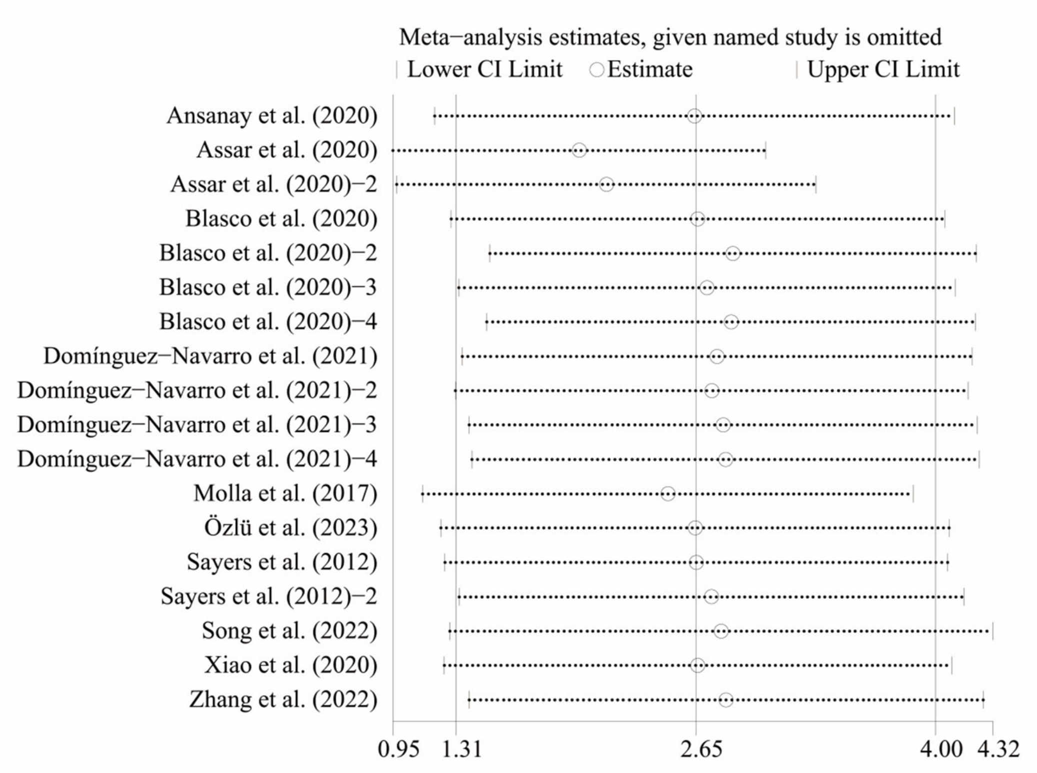

Figure S4. Sensitivity analysis of BBS.

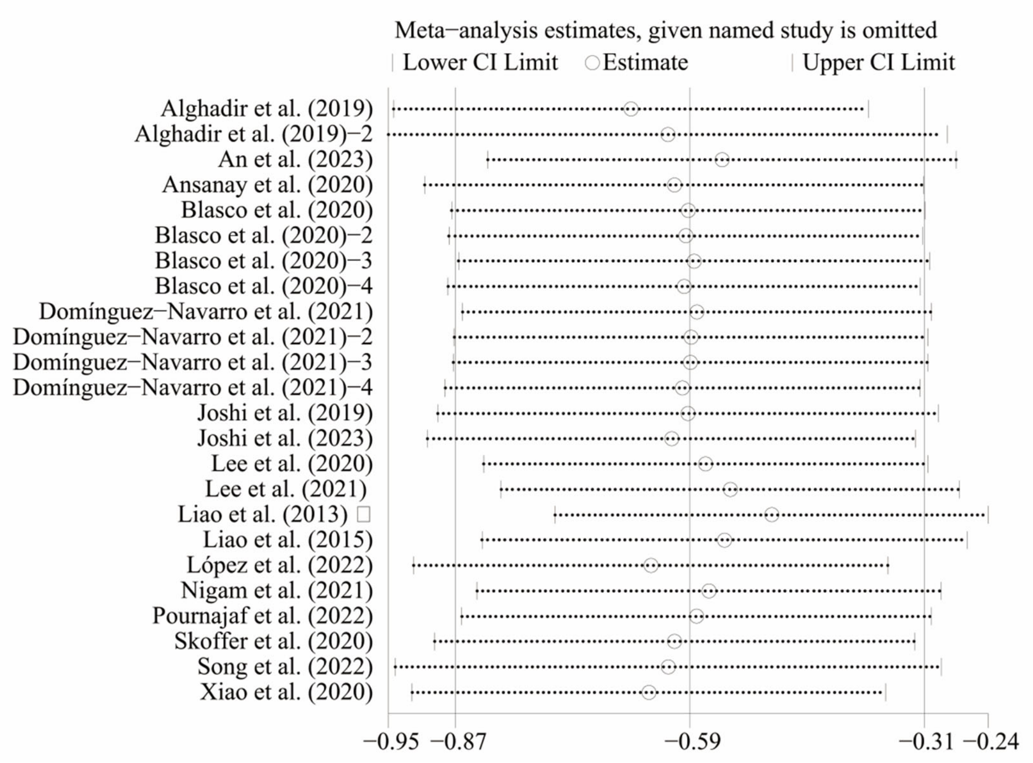

Figure S5. Sensitivity analysis of TUG.

Table S1. Search strategies.

Exercise search terms combined with “OR”
Physical exercise programs; Physical Therapy Modalities; Physical Therapy Modalities; Modalities, Physical Therapy; Modality, Physical Therapy; Physical Therapy Modality; Physiotherapy; Physical Therapy Techniques; Physical Therapy Technique; Techniques, Physical Therapy; Exercise Movement Techniques; Exercise Movement Techniques; Movement Techniques, Exercise; Exercise Therapy; Exercise Therapy; Therapy, Exercise; Exercise Therapies; Therapies, Exercise; Exercise, Physical; Exercises, Physical; Physical Exercise; Physical Exercises; Exercise, Isometric; Exercises, Isometric; Isometric Exercises; Isometric Exercise; Exercise, Aerobic; Aerobic Exercises; Aerobic Exercise; Resistance Training; Resistance Training; Training, Resistance; Strength Training; Training, Strength; Weight-Lifting Strengthening Program; Strengthening Program, Weight-Lifting; Strengthening Programs, Weight-Lifting; Weight Lifting Strengthening Program; Weight-Lifting Strengthening Programs; Weight-Lifting Exercise Program; Exercise Program, Weight-Lifting; Exercise Programs, Weight-Lifting; Weight Lifting Exercise Program; Weight-Lifting Exercise Programs; Weight-Bearing Strengthening Program; Strengthening Program, Weight-Bearing; Strengthening Programs, Weight-Bearing; Weight Bearing Strengthening Program; Weight-Bearing Strengthening Programs; Weight-Bearing Exercise Program; Exercise Program, Weight-Bearing; Exercise Programs, Weight-Bearing; Weight Bearing Exercise Program; Weight-Bearing Exercise Programs
**Knee osteoarthritis search terms combined with “OR”**
Osteoarthritis, Knee; Knee Osteoarthritides; Knee Osteoarthritis; Osteoarthritis of the Knee; Osteoarthritis of Knee
**Balance search terms combined with “OR”**
Balance; Postural Balance; Posture Equilibrium; Equilibrium, Posture; Posture Equilibriums; Balance, Postural; Postural Equilibrium; Equilibrium, Postural; Posture Balance; Balance, Posture; Posture Balances; Musculoskeletal Equilibrium; Equilibrium, Musculoskeletal; Postural Control; Control, Postural; Postural Controls; Posture Control; Control, Posture; Posture Controls

Table S2. Excluded studies list.

Reasons	Title
**Non-outcome indicators (*n* = 14)**	1. Randomized controlled trial of aquatic exercise for treatment of kneeosteoarthritis in elderly people<br>2. Efficacy of Baduanjin dance versus Thai boxing dance on clinical-related outcomes and balance ability among patients with knee osteoarthritis: A randomized, single-blinded comparative trial<br>3. The effect of strengthening and balance exercises on static stability indices in women with knee osteoarthritis: a randomized clinical trial<br>4. Efficacy of physiotherapy management of knee joint osteoarthritis: a randomised, double blind, placebo controlled trial<br>5. Preoperative high-intensity strength training improves postural control after TKA: randomized-controlled trial<br>6. Assessment of the impact of proprioceptive exercises on balance and proprioception in patients with advanced knee osteoarthritis<br>7. Therapeutic effect of whole body vibration on chronic knee osteoarthritis<br>8. Effects of increased standing balance on pain in patients with knee osteoarthritis<br>9. Effects of Tai Chi exercise on pain, balance, muscle strength, and perceived difficulties in physical functioning in older women with osteoarthritis: a randomized clinical trial<br>10. Effect of proximal exercises of lower extremity on static balance parameters during quiet standing<br>11. The effect of Wuqinxi exercises on the balance function and subjective quality of life in elderly, female knee osteoarthritis patients<br>12.Effects of Pilates training vs. suspension training on quality of life inwomen with knee osteoarthritis: a randomized controlled trial<br>13. The efficacy of balance and proprioception exercises in female patients with knee osteoarthritis: A randomized controlled study<br>14. Impacts of tai chi exercise on functional fitness in community-dwelling older adults with mild degenerative knee osteoarthritis: a randomized controlled clinical trial
**No data (*n* = 5)**	1. Effects of tai chi exercise on postural stability and control in elderly patients with knee osteoarthritis<br>2. Effectiveness of perturbation training in the treatment of osteoarthritis of the knee to improve balance and function<br>3. The effects of electrical stimulation combined with continuous passive motion versus isometric exercise on symptoms, functional capacity, quality of life and balance in knee osteoarthritis: randomized clinical trial<br>4. The effects of two different water exercise trainings on pain, functional status and balance in patients with knee osteoarthritis<br>5. Effects of biodex balance training on symptomatic knee osteoarthritis in Rawalpindi: a randomized control trial
**Full text not available (*n* = 3)**	1. Effects of otago exercise on postural balance and fear of falling in older fallers with knee osteoarthritis<br>2. Effects of resistance training on older women with knee osteoarthritis and total knee arthroplasty<br>3. Closed chain of motion therapy versus Wii-based external game therapy for the treatment of osteoarthritis of the knee: randomized controlled trial
**No control group data (*n* = 2)**	1. Intensive supervision of rehabilitation programme improves balance and functionality in the short term after bilateral total knee arthroplasty<br>2. Effect of a combination of whole body vibration exercise and squat training on body balance, muscle power, and walking ability in the elderly
**Non target group (*n* = 1)**	1. Comparative effects of 2 aqua exercise programs on physical function, balance, and perceived quality of life in older adults with osteoarthritis
**Non-exercise intervention (*n* = 1)**	1. The effect of transtheoretical model-lead intervention for knee osteoarthritis in older adults: a cluster randomized trial

Table S3. Characteristics of studies included in this meta-analysis.

Study	Sample Size (male/female)	Age (years)	BMI (kg/m^2)	Severity of KOA	Intervention	Session duration (min)	Frequency (times/week)	Intervention duration (week)	Balance indicators
Alghadir et al. (2019)	INT1: 21<br>INT2: 20<br>CON: 18	INT1: 54.6<br>INT2: 55.3<br>CON: 56.8	INT1: 26.2<br>INT2: 26.1<br>CON: 26.1	K/L degree 1-3	Aerobic exercise	20	3	6	TUG
An et al. (2023)	INT: 20 (F)<br>CON: 20 (F)	INT: 71.60 ± 3.53<br>CON: 70.04 ± 2.47	INT: 26.15 ± 2.89<br>CON: 25.92 ± 3.30	NR	Combined kinetic chain exercise program	30	5	4	TUG
Ansanay et al. (2020)	INT: 11 (2/9)<br>CON: 11 (5/6)	INT: 51.7 ± 7.7<br>CON: 51.7 ± 4.8	INT: 26.1 ± 4.7<br>CON: 26.2 ± 5.5	K/L degree 2-3	Resistance exercise	NR	2	4	BBS, TUG
Assar et al. (2020)	INT1: 12 (F)<br>INT2: 12 (F)<br>CON: 12 (F)	INT1: 57.5 ± 6.9<br>INT2: 55.9 ± 8.6<br>CON: 63.8 ± 7.5	INT1: 28.5 ± 3.7<br>INT2: 29.8 ± 7.2<br>CON: 23.1 ± 11.6	K/L degree ≥ 2	Aerobic exercise and resistance exercise	60, 90	3	8	BBS
Blasco et al. (2020)	INT1: 25 (6/19)<br>INT2: 26 (7/19)<br>CON:26 (11/15)	INT1: 70.2 ± 7.2<br>INT2: 72.3 ± 4.5<br>CON: 70.9 ± 9.5	INT1:32.5 ± 4.9<br>INT2: 30.8 ± 5.7<br>CON: 31.2 ± 4.6	NR	Lower limb strengthening exercises, balance-oriented training	45	3	2	BBS, TUG
Domínguez-Navarro et al. (2021)	INT1: 24 (10/14)<br>INT2: 20 (7/13)<br>CON: 21 (7/14)	INT1: 70.8 ± 5.4<br>INT2: 70.4 ± 6.4<br>CON: 70.2 ± 5.6	INT1: 70.8 ± 5.4<br>INT2: 70.4 ± 6.4<br>CON: 70.2 ± 5.6	K/L degree > 3	Progressive resistance exercise	30, 60	3	2,4	BBS, TUG
Joshi et al. (2019)	INT: 21 (12/9)<br>CON: 21 (8/13)	INT: 50.7 ± 7.3<br>CON: 54.3 ± 10.2	INT: 28.6 ± 10.2<br>CON: 28.1 ± 8.9	NR	Muscle strengthening exercises and retro walking training	25	3	6	TUG
Joshi et al. (2023)	INT: 28 (7/21)<br>CON: 26 (3/23)	INT: 58.07 ± 5.36<br>CON: 55.07 ± 7.10	INT: 26.34 ± 3.9<br>CON: 25.02 ± 3.80	K/L degree 2-3	Neuromuscular training	40	2	6	TUG

Lee et al. (2020)	INT: 15 (F)<br>CON: 15 (F)	INT: 70.13 ± 4.70<br>CON: 69.00 ± 6.44	INT: 24.69 ± 2.65<br>CON: 25.71 ± 4.10	NR	Visual feedback-based dynamic balance exercise	30	5	4	TUG
Lee et al. (2021)	INT: 19 (F)<br>CON: 19 (F)	INT: 72.05 ± 5.15<br>CON: 71.89 ± 5.44	INT: 25.27 ± 2.67<br>CON: 26.75 ± 4.03	NR	Dynamic balance training	30	5	6	TUG
Liao et al. (2013)	INT: 58 (12/46)<br>CON: 55 (18/37)	INT: 71.38 ± 6.57<br>CON: 72.94 ± 7.33	INT: 28.43 ± 4.00<br>CON: 26.91 ± 4.17	NR	Functional training and balance training	90	NR	8	TUG
Liao et al. (2015)	INT: 65 (16/49)<br>CON: 65 (23/42)	INT: 71.43 ± 6.33<br>CON: 73.40 ± 7.04	INT: 27.88 ± 5.02<br>CON: 26.97 ± 4.15	NR	Functional training and balance training	NR	3	8	TUG
López et al. (2022)	INT: 20 (F)<br>CON: 20 (F)	INT: 60.29 ± 9.91<br>CON: 65.18 ± 9.04	INT: 26.80 ± 5.75<br>CON: 25.36 ± 2.40	K/L degree ≥ 2	Aquatic training	NR	2	8	TUG
Molla et al. (2017)	INT: 20 (F)<br>CON: 20 (F)	INT: 69.4 ± 5.7<br>CON: 67.9 ± 5.3	INT: 30.2 ± 5.2<br>CON: 28.3 ± 5.3	K/L degree 4	Resistive exercises	60	3	6	BBS
Nigam et al. (2021)	INT: 20 (8/12)<br>CON: 20 (3/17)	INT: 58.5 ± 4.36<br>CON: 59.4 ± 6.57	INT: 26.2 ± 2.29<br>CON: 25.6 ± 1.83	K/L degree 1-3	Mobilisation with movement plus exercise	45	3	2	TUG
Özlü et al. (2023)	INT: 35 (18/17)<br>CON: 38 (12/26)	INT: 53.28 ± 10.42<br>CON: 53.71 ± 9.65	INT: 26.31 ± 4.13<br>CON: 27.12 ± 5.62	NR	VR program	15	5	3	BBS
Pournajaf et al. (2022)	INT: 29 (14/15)<br>CON: 27 (8/19)	INT: 68.31 ± 8.12<br>CON: 71.07 ± 5.75	INT: 28.51 ± 3.50<br>CON: 28.64 ± 4.79	NR	Balance training	45	5	3	TUG
Sayers et al. (2012)	INT1: 12 (9/3)<br>INT2: 10 (2/8)<br>CON: 11 (3/8)	INT1: 66.9 ± 4.9<br>INT2: 65.9 ± 8.3<br>CON: 68.4 ± 8.1	INT1: 28.4 ± 5.7<br>INT2: 33.1 ± 8.9<br>CON: 30.8 ± 6.8	NR	High-speed power training<br>Slow-speed power training	NR	3	12	BBS
Skoffer et al. (2020)	INT: 24 (8/16)<br>CON: 20 (9/11)	INT: 72.9 ± 6.0<br>CON: 70.9 ± 6.3	INT: 30.5<br>CON: 32.2	NR	Resistance exercise	NR	3	8	TUG

Song et al. (2022)	INT: 20 (F)<br>CON: 20 (F)	INT: 64.15 ± 8.56<br>CON: 64.15 ± 8.56	INT: 24.60 ± 5.64<br>CON: 24.37 ± 2.71	K/L degree 0-3	Improved Taichi movement	60	3	12	BBS, TUG
Xiao et al. (2020)	INT: 45<br>CON: 40	INT: 70.7 ± 9.36<br>CON: 70.2 ± 10.35	NC	K/L degree 1-2	Wuqinxi Qigong exercise	60	4	24	BBS, TUG
Zhang et al. (2022)	INT: 22<br>CON: 21	INT: 55.76 ± 8.37<br>CON: 53.40 ± 10.66	NC	K/L degree 1-2	Yijinjing Qigong exercise	40	2	12	BBS

Abbreviations: SD, standard deviation; INT, intervention group; CON, control group; VR, virtual reality; NR, no report; BBS, Berg balance scale; TUG, timed up and go test.

Table S4. Results of meta-regression.

Modalities	_ES	Coef.	Std. Err	t	P > |t|	95% CI
Intervention duration	subgroup	0.09139	0.18667	0.49	0.631	-0.3043421, 0.4871205
	_cons	2.16053	1.68891	1.28	0.219	-1.419811, 5.74086
Frequency	subgroup	0.31207	1.64401	0.19	0.852	-3.173067, 3.797216
	_cons	1.85691	5.12973	0.36	0.722	-9.017632, 12.73144
Session duration	subgroup	0.16804	0.06057	2.77	0.016	0.0371794, 0.2988947
	_cons	-5.05353	3.01571	-1.68	0.118	-11.56857, 1.461514
Weekly time	subgroup	0.04938	0.01895	2.61	0.022	-0.0084418, 0.0903258
	_cons	-4.28325	2.92260	-1.47	0.167	-10.59714, 2.030649
Participant age	subgroup	-0.27815	0.13071	-2.13	0.049	-0.5552589, -0.0010434
	_cons	20.93426	8.58745	2.44	0.027	2.72968, 39.13883

Abbreviations: Coef., coefficient; Std. Err., standard error; t, t-test statistic; CI, confidence interval.

Table S5. Results of Egger's test.

Measurements	Std_EFF	Coef.	Std. Err.	t	P > |t|	95% CI
**BBS**	Slope	-0.26393	1.56446	-0.17	0.868	-3.580434, 3.05258
	Bias	2.03050	1.28488	1.58	0.134	-0.6933363, 4.754325
**TUG**	Slope	-0.28333	0.11591	-2.44	0.023	-0.5237281, -0.0429461
	Bias	-0.72612	0.40126	-1.81	0.084	-1.558272, 0.106036

Abbreviations: BBS, Berg Balance Scale; TUG, Timed Up and Go test; Coef., coefficient; Std. Err., standard error; t, t-test statistic; CI, confidence interval.
